# Complications and adverse outcomes in pregnancy and childbirth among women who conceived by assisted reproductive technologies: a nationwide birth cohort study of Japan environment and children’s study

**DOI:** 10.1186/s12884-019-2213-y

**Published:** 2019-02-20

**Authors:** Chie Nagata, Limin Yang, Kiwako Yamamoto-Hanada, Hidetoshi Mezawa, Tadayuki Ayabe, Kazue Ishizuka, Mizuho Konishi, Yukihiro Ohya, Hirohisa Saito, Haruhiko Sago, Japan Environment & Children’s Study Group, Toshihiro Kawamoto, Reiko Kishi, Nobuo Yaegashi, Koichi Hashimoto, Chisato Mori, Shuichi Ito, Zentaro Yamagata, Hidekuni Inadera, Michihiro Kamijima, Takeo Nakayama, Hiroyasu Iso, Masayuki Shima, Yasuaki Hirooka, Narufumi Suganuma, Koichi Kusuhara, Takahiko Katoh

**Affiliations:** 1 Department of Education for Clinical Research, National Center for Child Health and Development, 2-10-1 Okura, Setagaya-ku, Tokyo, 157-8535 Japan; 2 Medical Support Center for Japan Environment and Children’s Study (JECS), National Center for Child Health and Development, 2-10-1 Okura, Setagaya-ku, Tokyo, 157-8535 Japan; 3 Division of Allergy, Department of Medical Subspecialties, National Center for Child Health and Development, 2-10-1 Okura, Setagaya-ku, Tokyo, 157-8535 Japan; 4 National Research Institute for Child Health and Development, National Center for Child Health and Development, 2-10-1 Okura, Setagaya-ku, Tokyo, 157-8535 Japan; 5 Center for Maternal-Fetal, Neonatal and Reproductive Medicine, National Center for Child Health and Development, 2-10-1 Okura, Setagaya-ku, Tokyo, 157-8535 Japan

**Keywords:** Assisted reproductive technology, ART, In vitro fertilization, IVF, Intracytoplasmic sperm injections, ICSI, Ovulation induction, Placental diseases, Blood transfusion, Intensive care unit

## Abstract

**Background:**

Although pregnancies conceived by assisted reproductive technology (ART) have a higher risk of maternal/perinatal complications, the overall risk of adverse outcomes necessitating advanced obstetric care has not been closely examined. The present study aimed to assess and compare the risk of maternal/perinatal complications and adverse outcomes in pregnancy and childbirth conceived by ART with those conceived naturally.

**Methods:**

This study was conducted as a part of the Japan environment and children’s study (JECS), an ongoing nationwide birth cohort study in Japan. The risk of maternal/perinatal complications and adverse outcomes was assessed by mode of conception (natural conception, ovulation induction [OI] without ART, conventional in vitro fertilization and embryo transfer [IVF-ET], or intracytoplasmic sperm injection [ICSI]) using logistic regression and generalized estimating equations controlling for potential confounders.

**Results:**

The final dataset included women who conceived naturally (*N* = 90,506), by OI without ART (*N* = 3939), by conventional IVF-ET (*N* = 1476), and by ICSI (*N* = 1671). Compared with women who conceived naturally, those who conceived by conventional IVF-ET were at higher risk of placenta previa (adjusted OR 2.90 [95% CI 1.94, 4.34]), morbidly adherent placenta (6.85 [3.88, 12.13]), and pregnancy-induced hypertension (1.40 [1.10, 1.78]) whereas those who conceived by ICSI had a higher risk of placental abruption (2.16 [1.20, 3.88]) as well as placenta previa (2.01 [1.29, 3.13]) and morbidly adherent placenta (7.81 [4.56, 13.38]). Women who conceived by ART had a higher risk of blood transfusion (conventional IVF-ET: 3.85 [2.52, 5.88]; ICSI: 3.76 [2.49, 5.66]) and ICU admission (conventional IVF-ET: 2.58 [1.11, 6.01]; ICSI: 3.45 [1.68, 7.06]) even after controlling for potential confounders. Neonates conceived by ART had a higher risk of preterm birth (conventional IVF-ET: 1.42 [1.13, 1.78]; ICSI: 1.31 [1.05, 1.64]).

**Conclusions:**

Women who conceived by ART had a higher risk of maternal/perinatal complications necessitating advanced obstetric care. Obstetricians should be aware of the increased risk of adverse outcomes among this population.

## Background

In recent years, a substantial number of children have been conceived using assisted reproductive technology (ART) particularly in high-income countries [1]. Currently the proportion of children conceived by ART in Japan is roughly 5% [2] and is rising [2–5]. However, women who conceive by ART have a higher risk of maternal and perinatal complications, such as pregnancy-induced hypertension (PIH), placenta previa, placental abruption, morbidly adherent placenta (MAP), preterm birth, and low birth weight [6–10]. Numerous studies have investigated the reasons for this higher risk without reaching any definite conclusions. Potential causes include underlying maternal characteristics which necessitated the use of ART as well as the ART itself [10–16].

Although complications related to ART pregnancies have been well studied using large-scale registry and cohort data worldwide, many of these studies have focused on outcomes in fetuses and neonates born after ART [7–9]. Less attention has been paid to adverse maternal outcomes, and only a few studies have assessed the overall risk of life-threatening conditions, such as blood transfusion, peripartum hysterectomy, intensive care unit (ICU) admission, and maternal death in this population [15–20]. In Japan, there is an online registration system which covers more than 90% of ART cycles conducted nationwide and their outcomes. Although this system collects detailed information on ART, data pertaining to maternal/perinatal complications and adverse outcomes are limited [2].

To redress this omission, we conducted the present study as an adjunct to the Japan environment and children’s study (JECS), a nationwide cohort study of environmental impacts on child health. The aim of the present study was to assess and compare the risk of maternal/perinatal complications and adverse outcomes between women who conceived by ART and women who conceived naturally.

## Methods

This study was conducted as an adjunct to the JECS, an ongoing nationwide birth cohort study in Japan aiming to determine the impact of environmental factors on child health (data set: jecs-ag-20,160,424). Details of the JECS protocol have been published elsewhere [21]. Briefly, JECS is being conducted in 15 regions covering a variety of regions throughout Japan. Expecting mothers were recruited either at cooperating obstetric facilities or local government offices between January 2011 and March 2014. JECS covers a total of 103,099 pregnancies and includes follow up studies of the children resulting from those pregnancies until they have reached the age of 13.

During pregnancy and at one month postpartum, various data were collected from the participants using a self-administered questionnaire. The collected information included maternal and paternal characteristics, anthropometric measurements, medical history, socio-economic status (e.g., income, occupation, and education), life-style (food consumption, exercise, and sleep), mental health, exposure to chemicals, etc. Furthermore, medical information pertaining to the pregnancy course, delivery, and postpartum condition was collected by extraction of data from the participants’ medical records either by clinicians or trained research coordinators.

JECS was approved by the institutional review board of the Ministry of the Environment and the ethics committees of all the participating institutions and is being conducted in accordance with the Declaration of Helsinki and other relevant regulations in Japan. Written informed consent was obtained from all the participants. As the present study used anonymized data, individual approval from the ethics committee was deemed unnecessary.

### Target population, variables of interest, and outcomes

The target population of the present study consisted of all women who participated in JECS and conceived by one of the following methods: 1) naturally; 2) using ovulation induction (OI) without ART; 3) conventional in vitro fertilization and embryo transfer (IVF-ET); or 4) intracytoplasmic sperm injection (ICSI). For the outcomes pertaining to fetuses/neonates, we analyzed only those delivered at 22 weeks or more of gestation. The variables of interest were conventional IVF-ET and ICSI. Women who conceived naturally served as the reference group. We also included women who conceived using OI without ART in order to estimate their risk level in case this might differ from that of natural pregnancies, given the former group’s history of infertility and the effect of OI [16, 17, 22].

Maternal complications and adverse outcomes included placenta previa, placental abruption, MAP, PIH, gestational diabetes, cesarean section, maternal blood transfusion, maternal admission to the ICU, and maternal death. Stillbirth (≥ 22 weeks of gestation), pre-term birth (< 37 weeks of gestation), and low birth weight (< 2500 g) were considered to be adverse fetal/neonatal outcomes. All of the maternal/perinatal complications and adverse outcomes were diagnosed following the protocol at each participating institution, which was presumably based on the relevant Japanese guidelines. Pregnancy-induced hypertension was defined as “hypertension (systolic blood pressure ≥140mmHg or diastolic blood pressure ≥90 mmHg) observed from the 20^th^ week of gestation to 12 weeks postpartum with or without proteinuria (≥300 mg/day), not just as a continuing pre-existing condition” [23]. Gestational diabetes was defined as a “glucose metabolism disorder occurring or recognized during pregnancy, excluding overt diabetes” [24]. An oral glucose tolerance test with 75 g sugar was used for diagnosis, and the diagnostic criteria were blood glucose values of: 1) ≥92 mg/dL in a fasted state; 2) ≥180 mg/dL after one hour; or 3) ≥153 mg/dL after two hours.

### Statistical analysis

First, we described the background characteristics of the participants who were included in the analysis, their pregnancy course, maternal and perinatal complications, and adverse outcomes by group (i.e., natural conception, OI without ART, conventional IVE-ET or ICSI). Next, the effect of each mode of conception on maternal/perinatal complications and adverse outcomes was assessed using logistic regression for maternal outcomes and generalized estimating equations (GEE) for fetal/neonatal outcomes, with women who conceived naturally serving as the reference group. The crude odds ratio (OR) and adjusted odds ratio (aOR) were calculated; adjusted covariates included maternal age, maternal body mass index (BMI) before pregnancy, maternal height, maternal weight before delivery, parity, prior cesarean section, pre-existing condition (e.g., chronic hypertension, hyperthyroidism, hypothyroidism, diabetes mellitus, autoimmune disease, heart disease, kidney disease, hepatitis, cerebral infarction, intracranial hemorrhage, epilepsy, blood disease, malignancy, psychiatric disorder, neurologic disease, thrombosis, and others), multiple pregnancies, fetal presentation, folic acid supplementation, maternal smoking during pregnancy, maternal drinking during pregnancy, maternal educational level, paternal smoking, paternal educational level, and household income. The variables included in the multivariate models as potential confounders were selected based on previous studies, biological plausibility, and their availability in the JECS data set [25–31]. We reported all crude ORs and aORs with the corresponding 95% confidence interval (CI). In order to improve the robustness of the analysis, we also conducted multiple imputation and compared the results with those from the model with case-wise deletion of missing data. The statistical analysis was conducted using the SAS software program (version 9.4; SAS Institute Inc., Cary, NC, USA).

## Results

**Fig. 1:**
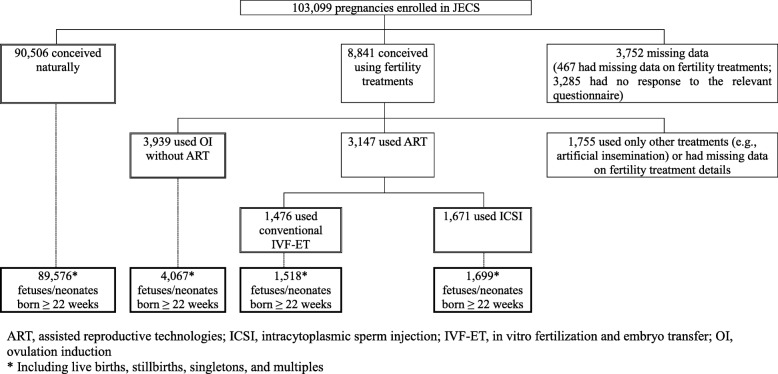
Flow chart of participants in the analysis

The final set of participants included in the main analysis consisted of women who conceived naturally (*N* = 90,506), by OI without ART (*N* = 3939), by conventional IVF-ET (*N* = 1476), and by ICSI (*N* = 1671). These pregnancies resulted in 96,860 fetuses/neonates in total delivered at 22 weeks or more of gestation, including live births and stillbirths, counting singletons and multiples, while excluding miscarriages/abortions before 22 weeks of gestation and cases with missing data on gestational age at birth. Figure 1 shows the flow chart of the participants who were either included in the main analysis or excluded for failing to meet the inclusion criteria.

Table 1 summarizes the background characteristics of the women who were included in the main analysis. Women who conceived by ART were more likely to be older, nulliparous, have a pre-existing condition, taking daily folic acid supplementation, non-smokers, have a higher education, and come from a wealthier household in contrast to women who conceived naturally. Table 2 shows the pregnancy and fetal/neonatal characteristics by mode of conception. Women who conceived by OI without ART had the highest proportion of multiple births among the four groups (4.9%). Table 3 shows the maternal/perinatal complications and adverse outcomes by mode of conception in all pregnancies, pregnancies with singletons, and pregnancies with multiples.

**Table 1:** Background characteristics of participants by mode of conception

	Natural conception		OI without ART		Conventional IVF-ET		ICSI	
	*N* = 90,506		*N* = 3939		*N* = 1476		*N* = 1671	
	Median	IQR	Median	IQR	Median	IQR	Median	IQR
Maternal age (years)	31	8	33	6	36	6	36	6
Missing (N)	733		31		4		17	
Maternal height (cm)	158.0	8.0	158.0	7.5	158.7	8.0	158.5	7.0
Missing (N)	477		23		3		6	
Maternal BMI before pregnancy (kg/m^2^)	20.6	3.5	20.6	3.7	20.7	3.4	20.7	3.3
Missing (N)	1089		41		15		17	
Maternal weight before delivery (kg)	62.3	10.6	62.1	11.1	62.4	10.6	61.8	10.5
Missing (N)	3019		158		55		79	
	N	%	N	%	N	%	N	%
Parity								
0	33,760	38.2	2290	59.9	949	66.0	1089	68.1
1	34,934	39.5	1331	34.8	447	31.1	469	29.3
2 or more	19,712	22.3	201	5.3	42	2.9	42	2.6
(Missing)	2100		117		38		71	
Prior cesarean section								
No	82,242	90.9	3680	93.5	1346	91.2	1530	91.6
Yes	8200	9.1	257	6.5	130	8.8	141	8.4
(Missing)	64		2		0		0	
Marital status								
Married	85,789	95.2	3919	99.6	1474	100.0	1668	99.8
Single	4304	4.8	16	0.4	0	0.0	3	0.2
(Missing)	413		4		2		0	
Pre-existing maternal condition								
No	74,963	85.4	3080	81.3	1087	75.3	1242	77.6
Yes	12,858	14.6	710	18.7	357	24.7	359	22.4
(Missing)	245		4		1		3	
Folic acid supplementation								
No	47,803	53.0	1310	33.3	470	31.9	476	28.5
A few times per month	4610	5.1	184	4.7	38	2.6	52	3.1
A few times per week	14,127	15.7	735	18.7	251	17.0	293	17.6
Daily	23,721	26.3	1706	43.4	716	48.5	847	50.8
(Missing)	695		21		8		7	
Maternal smoking during pregnancy								
No	85,152	94.8	3867	98.7	1453	99.0	1649	99.1
Yes	4659	5.2	51	1.3	15	1.0	15	0.9
(Missing)	440		10		3		3	
Maternal drinking during pregnancy								
No	81,033	90.0	3579	91.1	1362	92.5	1525	91.4
Yes	9033	10.0	350	8.9	111	7.5	143	8.6
(Missing)	2512		100		38		42	
Maternal educational level								
Junior high/high school	33,026	37.5	904	23.5	335	23.3	358	22.0
Vocational school/junior college	36,499	41.5	1866	48.6	673	46.8	777	47.7
University/graduate school	18,469	21.0	1069	27.8	430	29.9	494	30.3
(Missing)	1902		47		15		23	
Paternal smoking								
No	45,916	51.8	2464	63.3	977	66.9	1145	69.5
Yes	42,688	48.2	1428	36.7	484	33.1	503	30.5
(Missing)	3105		105		39		45	
Paternal educational level								
Junior high/high school	39,601	45.3	1229	32.1	475	33.1	503	30.9
Vocational school/junior college	19,687	22.5	902	23.5	272	18.9	351	21.6
University/graduate school	28,113	32.2	1703	44.4	690	48.0	772	47.5
(Missing)	8435		283		93		116	
Household income (×10,000 Japanese yen)								
< 200	4956	6.0	73	2.0	17	1.2	20	1.3
≥ 200, < 400	29,346	35.8	949	26.0	279	20.2	258	16.6
≥ 400, < 600	26,979	32.9	1319	36.1	480	34.7	521	33.5
≥ 600, < 800	12,554	15.3	747	20.4	318	23.0	391	25.1
≥ 800, < 1000	5012	6.1	328	9.0	161	11.6	204	13.1
≥ 1000	3224	3.9	240	6.6	128	9.3	161	10.4
(Missing)	8435		283		93		116	

*ART* assisted reproductive technologies, *BMI* body mass index, *ICSI* intracytoplasmic sperm injection. *IQR* interquartile range, *IVF-ET* in vitro fertilization and embryo transfer, *OI* ovulation induction

Proportions were calculated excluding cases with missing data

**Table 2:** Pregnancy and fetal/neonatal characteristics by mode of conception

	Natural conception		OI without ART		Conventional IVT-ET		ICSI	
	N	%	N	%	N	%	N	%
Pregnancies	*N* = 90,506		*N* = 3939		*N* = 1476		*N* = 1671	
Singleton pregnancy	88,873	99.3	3702	95.1	1407	95.7	1586	96.0
Multiple pregnancies	625	0.7	189	4.9	63	4.3	66	4.0
(Missing)	1008		48		6		19	
Fetus/Neonate born ≥22 weeks	*N* = 89,576		*N* = 4067		*N* = 1518		*N* = 1699	
Number of fetuses								
Singletons	88,337	98.6	3682	90.6	1393	91.8	1570	92.4
Multiples	1209	1.4	383	9.4	125	8.2	129	7.6
(Missing)	30		2		0		0	
Fetal presentation								
Cephalic	85,331	96.4	3726	93.2	1345	90.7	1516	90.8
Non-cephalic	3179	3.6	273	6.8	138	9.3	153	9.2
(Missing)	1066		68		35		30	
Sex of neonate								
Male	45,942	51.3	2062	50.7	805	53.0	850	50.0
Female	43,608	48.7	2003	49.3	713	47.0	849	50.0
(Missing)	26		2		0		0	

*ART* assisted reproductive technology, *ICSI* intracytoplasmic sperm injection, *ICU* intensive care unit, *IVF-ET* in vitro fertilization and embryo transfer, *OI* ovulation induction

Proportions were calculated excluding cases with missing data

**Table 3:** Maternal/perinatal complications and adverse outcomes by mode of conception in all pregnancies, pregnancies with singletons, and pregnancies with multiples

	Natural conception		OI without ART		Conventional IVT-ET		ICSI	
	N	%	N	%	N	%	N	%
All women and a fetus/neonate born ≥22 weeks								
Maternal outcomes	*N* = 90,506		*N* = 3939		*N* = 1476		*N* = 1671	
Placenta previa	489	0.5	22	0.6	36	2.4	30	1.8
Placental abruption	382	0.4	19	0.5	8	0.5	19	1.2
MAP	172	0.2	9	0.2	17	1.2	24	1.5
Gestational diabetes	2304	2.6	138	3.5	76	5.2	80	4.8
PIH	2632	2.9	177	4.5	101	6.9	107	6.5
Cesarean section	16,433	18.5	933	24.2	563	38.6	617	37.8
Blood transfusion	377	0.4	20	0.5	36	2.4	38	2.3
ICU admission	105	0.1	8	0.2	7	0.5	15	0.9
Maternal death	10	0	0	0	0	0	0	0
Fetal/neonatal outcomes	*N* = 89,576		*N* = 4067		*N* = 1518		*N* = 1699	
Stillbirth	230	0.3	17	0.4	10	0.7	5	0.3
Preterm birth < 37 w	4768	5.3	423	10.4	184	12.1	181	10.7
Low birth weight < 2500 g	7980	8.9	651	16.0	243	16.0	245	14.4
Women with a singleton pregnancy and singleton born ≥22 weeks								
Maternal outcomes	*N* = 88,873		*N* = 3702		*N* = 1407		*N* = 1586	
Placenta previa	486	0.5	20	0.5	34	2.4	28	1.8
Placental abruption	380	0.4	18	0.5	8	0.6	19	1.2
MAP	172	0.2	8	0.2	17	1.2	24	1.5
Gestational diabetes	2271	2.6	132	3.6	72	5.1	75	4.7
PIH	2573	2.9	165	4.5	93	6.6	102	6.4
Cesarean section	15,912	18.0	780	21.2	509	36.5	558	35.6
Blood transfusion	368	0.4	16	0.4	33	2.4	37	2.3
ICU admission	97	0.1	5	0.1	7	0.5	12	0.8
Maternal death	10	0	0	0	0	0	0	0
Fetal/neonatal outcomes	*N* = 88,337		*N* = 3682		*N* = 1393		*N* = 1570	
Stillbirth	216	0.2	8	0.2	8	0.6	4	0.3
Preterm birth	4153	4.7	211	5.7	120	8.6	119	7.6
Low birth weight < 2500 g	7141	8.1	373	10.1	159	11.4	159	10.1
Women with multiple pregnancies and multiples born ≥22 weeks								
Maternal outcomes	*N* = 625		*N* = 189		*N* = 63		*N* = 66	
Placenta previa	3	0.5	1	0.5	2	3.2	2	3.1
Placental abruption	2	0.3	1	0.5	0	0	0	0
MAP	0	0	1	0.5	0	0	0	0
Gestational diabetes	31	5.1	6	3.2	4	6.3	5	7.8
PIH	57	9.4	12	6.5	8	12.7	5	7.8
Cesarean section	518	85.8	151	81.2	54	87.1	59	92.2
Blood transfusion	9	1.5	3	1.6	3	4.8	1	1.6
ICU admission	8	1.3	3	1.6	0	0	3	4.7
Maternal death	0	0	0	0	0	0	0	0
Fetal/neonatal outcomes	*N* = 1209		*N* = 383		*N* = 125		*N* = 129	
Stillbirth	13	1.1	9	2.3	2	1.6	1	0.8
Preterm birth	613	50.7	212	55.4	64	51.2	62	48.1
Low birth weight < 2500 g	838	69.3	278	72.8	84	67.2	86	66.7

*ART* assisted reproductive technologies, *ICSI* intracytoplasmic sperm injection, *ICU* intensive care unit, *IVF-ET* in vitro fertilization and embryo transfer, *MAP* morbidly adherent placenta, *OI* ovulation induction, *PIH* pregnancy-induced hypertension

Proportions were calculated excluding cases with missing data

The association between each mode of conception and maternal/perinatal complications and adverse outcomes was assessed using a logistic regression and GEE analysis, with women who conceived naturally serving as a reference (Table 4). Pregnancies resulting from OI without ART did not have an increased risk of maternal/perinatal complications or adverse outcomes. Maternal death and stillbirth were not assessed because the number of cases was insufficient to calculate the aORs.

**Table 4:** Effect of mode of conception on maternal/perinatal complications and adverse outcomes

	Logistic regression with case-wise deletion of missing data						Multiple imputation		
	Crude OR	95% CI		aOR	95% CI		aOR	95% CI	
OI without ART									
Maternal outcomes^a^									
Placenta previa	0.76	0.43	1.31	0.62	0.35	1.09	0.85	0.55	1.32
Placental abruption	1.33	0.82	2.18	1.33	0.80	2.19	1.13	0.71	1.81
MAP	1.46	0.74	2.86	1.46	0.73	2.91	1.23	0.62	2.45
Gestational diabetes	1.40	1.16	1.69	0.97	0.80	1.19	0.98	0.81	1.18
PIH	1.55	1.30	1.84	1.10	0.91	1.32	1.11	0.94	1.31
Cesarean section	1.41	1.29	1.53	1.12	0.99	1.26	1.08	0.97	1.21
Blood transfusion	1.35	0.83	2.21	1.03	0.62	1.71	0.94	0.59	1.5
ICU admission	2.16	1.04	4.46	1.29	0.60	2.79	1.07	0.49	2.3
Fetal/neonatal outcomes^b^									
Preterm birth	1.93	1.71	2.18	1.05	0.09	1.24	1.01	0.86	1.18
Low birth weight	1.88	1.70	2.07	1.04	0.93	1.19	1.04	0.93	1.17
Conventional IVT-ET									
Maternal outcomes^a^									
Placenta previa	4.68	3.22	6.82	2.90	1.94	4.34	2.86	1.99	4.12
Placental abruption	1.03	0.43	2.50	0.91	0.37	2.26	1.08	0.53	2.22
MAP	6.88	4.09	11.58	6.85	3.88	12.13	5.74	2.93	11.2
Gestational diabetes	1.88	1.45	2.45	0.99	0.75	1.31	1.13	0.88	1.45
PIH	2.43	1.94	3.05	1.40	1.10	1.78	1.41	1.13	1.76
Cesarean section	2.69	2.39	3.02	1.85	1.58	2.17	1.9	1.64	2.19
Blood transfusion	6.18	4.20	9.09	3.85	2.52	5.88	3.57	2.45	5.21
ICU admission	4.98	2.30	10.80	2.58	1.11	6.01	2.04	0.89	4.66
Fetal/neonatal outcomes^b^									
Preterm birth	2.59	2.18	3.08	1.42	1.13	1.78	1.36	1.11	1.66
Low birth weight	1.90	1.63	2.22	0.94	0.76	1.15	1.01	0.85	1.2
ICSI									
Maternal outcomes^a^									
Placenta previa	3.37	2.22	5.11	2.01	1.29	3.13	2.02	1.36	3.01
Placental abruption	2.44	1.40	4.26	2.16	1.20	3.88	2.35	1.44	3.82
MAP	7.41	4.57	12.00	7.81	4.56	13.38	7.86	4.56	13.5
Gestational diabetes	1.85	1.43	2.38	0.97	0.74	1.27	1.06	0.83	1.35
PIH	2.16	1.73	2.71	1.25	0.98	1.60	1.34	1.07	1.67
Cesarean section	2.75	2.46	3.07	1.89	1.62	2.19	1.82	1.59	2.09
Blood transfusion	5.98	4.11	8.69	3.76	2.49	5.66	3.43	2.37	4.96
ICU admission	7.11	3.78	13.36	3.45	1.68	7.06	3.87	2.07	7.24
Fetal/neonatal outcomes^b^									
Preterm birth	2.33	1.96	2.77	1.31	1.05	1.64	1.21	0.99	1.47
Low birth weight	1.78	1.53	2.07	0.87	0.72	1.06	0.89	0.74	1.06

*aOR* adjusted odds ratio, *ART* assisted reproductive technologies, *CI* confidence intervals, *ICSI* intracytoplasmic sperm injection, *ICU* intensive care unit, *IVF-ET* in vitro fertilization and embryo transfer, *MAP* morbidly adherent placenta, *OI* ovulation induction, *OR* odds ratio, *PIH* pregnancy-induced hypertension

Women who conceived naturally served as the reference group

Adjusted odds ratios were calculated by controlling for maternal age, maternal body mass index (BMI) before pregnancy, maternal height, maternal weight before delivery, parity, prior cesarean section, pre-existing maternal conditions, multiple pregnancies, fetal presentation, folic acid supplementation, maternal smoking during pregnancy, maternal drinking during pregnancy, maternal educational level, paternal smoking, paternal educational level, and household income

^a^logistic regression; ^b^generalized estimating equations (GEE)

Women who conceived by conventional IVF-ET were at higher risk of placenta previa (aOR 2.90 [95% CI 1.94, 4.34]), MAP (aOR 6.85 [95% CI 3.88, 12.13]), and PIH (aOR 1.40 [95% CI 1.10, 1.78]) than those who conceived naturally. Women who conceived by ICSI had a higher risk of placental abruption (aOR 2.16 [95% CI 1.20, 3.88]) as well as placenta previa (aOR 2.01 [95% CI 1.29, 3.13]), MAP (aOR 7.81 [95% CI 4.56, 13.38]), and marginally significant PIH (aOR 1.25 [95% CI 0.98, 1.60]). Furthermore, women who conceived by ART were more likely to have a cesarean section and had a significantly higher risk of blood transfusion (conventional IVF-ET: aOR 3.85 [95% CI 2.52, 5.88]; ICSI: aOR 3.76 [95% CI 2.49, 5.66]) and ICU admission (conventional IVF-ET: aOR 2.58 [95% CI 1.11, 6.01]; ICSI: aOR 3.45 [95% CI 1.68, 7.06]) even after controlling for potential confounders. Neonates born after ART were at higher risk of preterm birth (conventional IVF-ET: aOR 1.42 [95% CI 1.13, 1.78]; ICSI: aOR 1.31 [95% CI 1.05, 1.64]) compared to those conceived naturally.

We also performed multiple imputation and compared the results with those from the logistic regression model with case-wise deletion of missing data (Table 4). The estimated effects of the variables of interest were similar in terms of their direction and magnitude.

## Discussion

Compared with women who conceived naturally, those who conceived by conventional IVF-ET were at higher risk of placenta previa, MAP, and PIH whereas those who conceived by ICSI had a higher risk of placental abruption in addition to the above. Women who conceived by ART had a significantly higher risk of blood transfusion and ICU admission even after controlling for maternal age, pre-existing condition, and other potential confounders. Neonates conceived by ART were at higher risk of preterm birth.

The increased risk of maternal and perinatal complications observed in the present study were largely consistent with the findings of previous studies [6–10, 14–16]. Among these complications, the risk of MAP was conspicuous, with an aOR of 6.85 for conventional IVF-ET and 7.81 for ICSI. More than 1% of women who conceived by ART had MAP; however, this figure should be interpreted with caution as the diagnoses were made clinically irrespective of pathological examination in JECS. The relationship between IVF pregnancies and MAP was first reported by Esh-Broder, et al. in 2011 [10]. MAP is one of the major causes of catastrophic outcomes in obstetrics, and placenta previa and previous cesarean section are known risk factors of MAP [32, 33]. Prenatal diagnosis is crucial for appropriate management, and ultrasound examination and magnetic resonance imaging are used especially for women with risk factors [34, 35]. However, prenatal diagnosis is not always possible. Given the observed relationship between ART and MAP, obstetricians should consider women who conceived by ART as a high risk group for MAP regardless of their prenatal diagnosis. In addition to the incidence of MAP, the observed incidence of placenta previa was also high among ART pregnancies (2.4% for conventional IVF, 1.8% for ICSI).

With regard to life-threatening maternal conditions, studies such as the one by Belanoff et al. have reported a higher risk of severe maternal morbidity in women who conceived using ART [17–19]. Cromi et al. reported on the risk of a peripartum hysterectomy in pregnancies resulting from ART in 2016, arguing that such pregnancies should be managed as “high risk” [20]. In our study, more than 2% of women who conceived by ART received a blood transfusion. We realize that the incidence of these complications may be overestimated (or underestimated) depending upon the participants’ characteristics, given that JECS is not completely population-based. However, according to the profile paper of JECS, characteristics of the women and children who participated in JECS appeared to be comparable to those reported in Japan’s Vital Statistics Survey [36]. Hence, the observed higher risk of blood transfusion (conventional IVF-ET: aOR 3.85, ICSI: aOR 3.76) and ICU admission (conventional IVF-ET: aOR 2.58, ICSI: aOR 3.45) in ART pregnancies is deemed to be reliable.

In the present study, we estimated the risk levels of women who conceived using OI without ART separately from those of women who conceived naturally, considering the possibility that they might differ given the former’s history of infertility and the effect of OI. Although univariate analysis demonstrated an increased risk for some of the maternal/perinatal complications and adverse outcomes, none reached statistical significance after controlling for potential confounders.

The present study has several strengths. First and foremost, JECS is the largest birth cohort in the country and collects wide-ranging, in-depth information from participants and their medical records. This enabled us to control for various potential confounders including participants’ socio-economic status. In addition, the present study included blood transfusion and ICU admission as maternal adverse outcomes, unlike previous studies.

Nonetheless, the present study has several limitations. First, JECS was not designed to cover all expecting mothers during the recruitment period or apply complete random sampling (JECS aimed to cover 50% of the births in each study area [21].) Therefore, bias may have entered into the selection of the participants, thus leading to overestimation or underestimation of the incidence of maternal/perinatal complications and adverse outcomes. As mentioned above, however, the JECS profile paper suggested that the characteristics of the study participants were comparable to those collected in the national survey [36]. Second, the data pertaining to each woman’s mode of conception were self-reported, potentially jeopardizing their reliability. Furthermore, detailed data on the drugs and techniques used for OI or ART (e.g., fresh or frozen embryo transfer, blastocyst or cleavage stage embryo transfer) were not assessed. Third, data on some of the adverse outcomes, such as hysterectomy, were not collected in JECS. The number of maternal deaths and stillbirths was limited; hence, these figures were not included in the main analysis. Last, the diagnoses of maternal and perinatal complications were made at each participating facility, and subsequent interventions (e.g., blood transfusion and ICU admission) were conducted based on institutional protocols; hence, there may have been some variation in the diagnostic criteria and/or management strategies.

## Conclusions

Women who conceived by ART were at higher risk of maternal and perinatal complications necessitating advanced/emergency obstetric care, such as a blood transfusion or ICU admission. Obstetricians should be aware of the increased risk of adverse maternal outcomes among women who conceived by ART.

## Abbreviations

aOR: Adjusted odds ratio
ART: Assisted reproductive technology
BMI: Body mass index
CI: Confidence interval
ICSI: Intracytoplasmic sperm injection
ICU: Intensive care unit
IQR: Interquartile range
IVF-ET: In vitro fertilization and embryo transfer
JECS: Japan Environment and Children’s Study
MAP: Morbidly adherent placenta
NA: Not available
OI: Ovulation induction
OR: Odds ratio
PIH: Pregnancy-induced hypertension

## References

[1] Dyer S, Chambers GM, de Mouzon J, Nygren KG, Zegers-Hochschild F, Mansour R (2016). International committee for monitoring assisted reproductive technologies world report: assisted reproductive technology 2008, 2009 and 2010. Hum Reprod.

[2] Report from Registration and Investigation Subcommittee 2015. Registration and Investigation Subcommittee, Japan Society of Obstetrics and Gynecology. Acta Obst Gynaec Jpn. 2017;69:1841–915. (Japanese).

[3] Report from Registration and Investigation Subcommittee 2014. Registration and Investigation Subcommittee, Japan Society of Obstetrics and Gynecology. Acta Obst Gynaec Jpn. 2016;68:2077–2122. (Japanese).

[4] Report from Registration and Investigation Subcommittee 2013. Registration and Investigation Subcommittee, Japan Society of Obstetrics and Gynecology. Acta Obst Gynaec Jpn. 2015;67:2077–2121. (Japanese).

[5] Takeshima K, Saito H, Nakaza A, Kuwahara A, Ishihara O, Irahara M (2014). Efficacy, safety, and trends in assisted reproductive technology in Japan-analysis of four-year data from the national registry system. J Assist Reprod Genet.

[6] Vermey BG, Buchanan A, Chambers GM, Kolibianakis EM, Bosdou J, Chapman MG (2018). Are singleton pregnancies after assisted reproduction technology (ART) associated with a higher risk of placental anomalies compared with non-ART singleton pregnancies?.

[7] Qin J, Liu X, Sheng X, Wang H, Gao S (2016). Assisted reproductive technology and the risk of pregnancy-related complications and adverse pregnancy outcomes in singleton pregnancies: a meta-analysis of cohort studies. Fertil Steril.

[8] Qin J, Wang H, Sheng X, Liang D, Tan H, Xia J (2015). Pregnancy-related complications and adverse pregnancy outcomes in multiple pregnancies resulting from assisted reproductive technology: a meta-analysis of cohort studies. Fertil Steril.

[9] Pandey S, Shetty A, Hamilton M, Bhattacharya S, Maheshwari A (2012). Obstetric and perinatal outcomes in singleton pregnancies resulting from IVF/ICSI: a systematic review and meta-analysis. Hum Reprod Update.

[10] Esh-Broder E, Ariel I, Abas-Bashir N, Bdolah Y, Celnikier DH (2011). Placenta accreta is associated with IVF pregnancies: a retrospective chart review. BJOG.

[11] Pinborg A, Wennerholm UB, Romundstad LB, Loft A, Aittomaki K, Soderstrom-Anttila V (2013). Why do singletons conceived after assisted reproduction technology have adverse perinatal outcome? Systematic review and meta-analysis. Hum Reprod Update.

[12] Sazonova A, Kallen K, Thurin-Kjellberg A, Wennerholm UB, Bergh C (2011). Factors affecting obstetric outcome of singletons born after IVF. Hum Reprod.

[13] Romundstad LB, Romundstad PR, Sunde A, von During V, Skjaerven R, Gunnell D (2008). Effects of technology or maternal factors on perinatal outcome after assisted fertilisation: a population-based cohort study. Lancet.

[14] Romundstad LB, Romundstad PR, Sunde A, von During V, Skjaerven R, Vatten LJ (2006). Increased risk of placenta previa in pregnancies following IVF/ICSI; a comparison of ART and non-ART pregnancies in the same mother. Hum Reprod.

[15] Healy DL, Breheny S, Halliday J, Jaques A, Rushford D, Garrett C (2010). Prevalence and risk factors for obstetric haemorrhage in 6730 singleton births after assisted reproductive technology in Victoria Australia. Hum Reprod.

[16] Hayashi M, Nakai A, Satoh S, Matsuda Y (2012). Adverse obstetric and perinatal outcomes of singleton pregnancies may be related to maternal factors associated with infertility rather than the type of assisted reproductive technology procedure used. Fertil Steril.

[17] Belanoff C, Declercq ER, Diop H, Gopal D, Kotelchuck M, Luke B (2016). Severe maternal morbidity and the use of assisted reproductive Technology in Massachusetts. Obstet Gynecol.

[18] Wang ET, Ozimek JA, Greene N, Ramos L, Vyas N, Kilpatrick SJ (2016). Impact of fertility treatment on severe maternal morbidity. Fertil Steril.

[19] Martin AS, Monsour M, Kissin DM, Jamieson DJ, Callaghan WM, Boulet SL (2016). Trends in severe maternal morbidity after assisted reproductive Technology in the United States, 2008-2012. Obstet Gynecol.

[20] Cromi A, Candeloro I, Marconi N, Casarin J, Serati M, Agosti M (2016). Risk of peripartum hysterectomy in births after assisted reproductive technology. Fertil Steril.

[21] Kawamoto T, Nitta H, Murata K, Toda E, Tsukamoto N, Hasegawa M (2014). Rationale and study design of the Japan environment and children's study (JECS). BMC Public Health.

[22] Jaques AM, Amor DJ, Baker HW, Healy DL, Ukoumunne OC, Breheny S (2010). Adverse obstetric and perinatal outcomes in subfertile women conceiving without assisted reproductive technologies. Fertil Steril.

[23] Best practice guide for pregnancy induced hypertension 2015. Japan Society for the Study of Hypertension in Pregnancy. Tokyo: Medical View co., ltd; 2015:28–32. (Japanese).

[24] Gestational diabetes mellitus, In Practice and management manual for glucose metabolism disorder in pregnant women. The Japanese Society of Diabetes and Pregnancy. Tokyo: Medical View co., ltd; 2015:39–41. (Japanese).

[25] Ogawa K, Urayama KY, Tanigaki S, Sago H, Sato S, Saito S (2017). Association between very advanced maternal age and adverse pregnancy outcomes: a cross sectional Japanese study. BMC Pregnancy Childbirth.

[26] Marshall NE, Biel FM, Boone-Heinonen J, Dukhovny D, Caughey AB, Snowden JM. The Association between Maternal Height, Body Mass Index, and Perinatal Outcomes. Am J Perinatol. 2018. 10.1055/s-0038-1673395. 10.1055/s-0038-1673395 PMC6453733 30292175

[27] Morisaki N, Nagata C, Jwa SC, Sago H, Saito S, Oken E (2017). Pre-pregnancy BMI-specific optimal gestational weight gain for women in Japan. J Epidemiol.

[28] Fujiwara T, Ito J, Kawachi I (2013). Income inequality, parental socioeconomic status, and birth outcomes in Japan. Am J Epidemiol.

[29] Lassi ZS, Salam RA, Haider BA, Bhutta ZA. Folic acid supplementation during pregnancy for maternal health and pregnancy outcomes. Cochrane Database Syst Rev. 2013;(3):Cd006896. 10.1002/14651858.CD006896.pub2 PMC10069458 23543547

[30] Miyake Y, Tanaka K, Okubo H, Sasaki S, Arakawa M (2014). Alcohol consumption during pregnancy and birth outcomes: the Kyushu Okinawa maternal and child health study. BMC Pregnancy Childbirth..

[31] Miyake Y, Tanaka K, Arakawa M (2013). Active and passive maternal smoking during pregnancy and birth outcomes: the Kyushu Okinawa maternal and child health study. BMC Pregnancy Childbirth..

[32] Thurn L, Lindqvist PG, Jakobsson M, Colmorn LB, Klungsoyr K, Bjarnadottir RI (2016). Abnormally invasive placenta-prevalence, risk factors and antenatal suspicion: results from a large population-based pregnancy cohort study in the Nordic countries. BJOG.

[33] Bowman ZS, Eller AG, Bardsley TR, Greene T, Varner MW, Silver RM (2014). Risk factors for placenta accreta: a large prospective cohort. Am J Perinatol.

[34] Riteau AS, Tassin M, Chambon G, Le Vaillant C, de Laveaucoupet J, Quere MP (2014). Accuracy of ultrasonography and magnetic resonance imaging in the diagnosis of placenta accreta. PLoS One.

[35] Bowman ZS, Eller AG, Kennedy AM, Richards DS, Winter TC, 3rd, Woodward PJ, et al. Accuracy of ultrasound for the prediction of placenta accreta. Am J Obstet Gynecol. 2014;211(2):177.e171–e177. 10.1016/j.ajog.2014.03.029 24631709

[36] Michikawa T, Nitta H, Nakayama SF, Yamazaki S, Isobe T, Tamura K (2017). Baseline profile of participants in the Japan environment and Children's study (JECS). J Epidemiol..

